# KIAA1429 in non-small cell lung cancer: bridging m6A epigenetics to therapeutic innovation

**DOI:** 10.3389/fsurg.2025.1643120

**Published:** 2025-10-20

**Authors:** Hui Liu, Ke Shi, Liang Liu, Bo-Hua You, Tao Liu, Xiao-Fei Ren, Qiang Guo, Dan Li

**Affiliations:** 1 Department of Ultrasound Medicine, Taihe Hospital, Hubei University of Medicine, Shiyan, China; 2 Department of Thoracic Surgery, Beilun District People’s Hospital of Ningbo, Ningbo, China; 3 Department of Thoracic Surgery, Renmin Hospital of Wuhan University, Wuhan, China; 4 Department of Cardiothoracic Surgery, Taikang Tongji (Wuhan) Hospital, Wuhan, China; 5 Department of Thoracic Surgery, The People's Hospital of Honghu, Jingzhou, China; 6 Department of Cardiothoracic Surgery, Taihe Hospital, Hubei University of Medicine, Shiyan, China; 7 Department of Oncology, Taihe Hospital, Hubei University of Medicine, Shiyan, China

**Keywords:** KIAA1429, m6A modification, non-small cell lung cancer, molecular target, gefitinib resistance

## Abstract

As a pivotal component of the m6A methyltransferase complex, KIAA1429 plays a critical regulatory role in the pathogenesis of non-small cell lung cancer (NSCLC), driving tumorigenesis, metastasis, and therapeutic resistance through epigenetic mechanisms. Clinically, KIAA1429 overexpression correlates with aggressive disease progression and poor patient prognosis to conventional therapies. This review comprehensively examines the dysregulated expression patterns and functions of KIAA1429 in NSCLC, elucidating its m6A-dependent modulation of key downstream effectors (Such as the HOXA1, DAPK3, and BTG2) that orchestrate malignant transformation. We highlight the emerging potential of KIAA1429 as a novel molecular target for precision therapy in NSCLC.

## 1 Introduction

As is widely recognized, non-small cell lung cancer (NSCLC) represents one of the most prevalent malignancies worldwide, with a persistently low 5-year survival rate among patients (1–3). Consequently, improving the prognosis of cancer patients remains a key focus in oncology research. In recent years, targeted therapy has emerged as a crucial treatment modality for NSCLC patients (4–6). Notably, Hishida et al., reported a study involving 14 patients who underwent pulmonary resection following systemic therapy. Among them, 8 patients received epidermal growth factor receptor tyrosine kinase inhibitors (EGFR-TKIs), while 6 did not. With a median follow-up of 5 years, the EGFR-TKI group demonstrated a significantly higher 5-year overall survival (OS) rate of 83% compared to a mere 33% 5-year recurrence-free survival rate in the non-EGFR-TKI group (4). However, the development of EGFR-TKI resistance has become a major clinical challenge, serving as a primary cause of tumor recurrence or progression in NSCLC patients. Recent studies have revealed the critical involvement of N6-methyladenosine (m6A)-related genes in cancer proliferation and metastasis (7–17). For instance, Li et al., demonstrated that insulin-like growth factor 2 mRNA-binding protein 2 (IGF2BP2) is upregulated in lung adenocarcinoma (LUAD) cells. Their findings indicated that IGF2BP2 overexpression enhances solute carrier family 7 member 11 (SLC7A11) mRNA stability through m6A modification, thereby promoting cell viability and suppressing ferroptosis (14). Specifically, BRAF mutations can affect KIAA1429's localization within cells, leading to increased cytoplasmic expression and enhanced resistance to chemotherapy in colorectal cancer (17). KIAA1429, a core component of the m6A methyltransferase complex, has been shown to play a pivotal role in cell metastasis, proliferation, and drug resistance (18–31). For instance, Xu et al., demonstrated that KIAA1429 is significantly upregulated in LUAD tissues and cell lines. KIAA1429 promotes LINC01106 expression through m6A modification, ultimately enhancing tumor cell proliferation, invasion, migration, and xenograft tumor growth in nude mice (18, 21). In addition, Zhao et al., reported that elevated KIAA1429 expression correlates with clinically aggressive features in LUAD patients, including larger tumor diameter, lymph node metastasis, advanced disease stage, and poorer overall survival. Their work further elucidated that KIAA1429 knockdown suppresses MUC3A expression, thereby inhibiting LUAD cell proliferation, migration, invasion, and inducing cell cycle arrest (21). In light of these discoveries, this review systematically examines the expression patterns, functional significance, and clinical implications of KIAA1429 in NSCLC. It elucidates the molecular mechanisms by which KIAA1429 promotes malignant phenotypes through m6A-dependent regulation of target genes, while also evaluating its potential as a novel therapeutic target for NSCLC treatment.

## 2 Expression profile of KIAA1429 and its association with prognosis in NSCLC patients

Aberrant expression of KIAA1429 has been linked to poor OS in various cancers (32–35), and KIAA1429 is significantly overexpressed in both NSCLC tissues and cell lines (Table 1). For example, Xu et al., demonstrated that KIAA1429 is highly expressed in NSCLC tissues, particularly in the LUAD subtype. Compared to normal bronchial epithelial cells (HBE), KIAA1429 levels were markedly elevated in multiple NSCLC cell lines, including the A549, H1299, 95-D, NCI-H460, PC9, PC9GR, LTEP-a-2, and H520 cells. Similarly, when compared to the normal lung epithelial cell line (BEAS-2B), KIAA1429 expression was significantly higher in NSCLC SK-MES-1, H520, H2228, A549, H1299, HCC827, and PC9 cells (Table 1). In addition, KIAA1429 overexpression correlates with shorter OS in both NSCLC and LUAD patients. Additionally, high KIAA1429 expression is associated with the advanced pathological stage in both NSCLC and LUAD patients, and associated with the smoking history, larger tumor size, lymph node metastasis, distant metastasis, higher T-stage, and tumor invasion depth in LUAD patients (Table 2). These findings suggest that KIAA1429 may serve as a potential prognostic biomarker and contribute to tumor aggressiveness in NSCLC.

**Table 1:** The overexpression of KIAA1429 in NSCLC tissues and cells.

Authors	Type	Expression in tissues	*N*	Expression in cells	Cancer cell lines	Relative cells	Ref
Xu 2024 et al.,	LUAD	Overexpression	48	-	-	-	(18)
Guo 2024 et al.,	LUAD	Overexpression	80	-	-	-	(19)
Lin 2023 et al.,	LUAD	Overexpression	128	-	-	-	(20)
Zhao 2020 et al.,	LUAD	Overexpression	50	Overexpression	A549, H1299, 95-D, NCI-H460	HBE	(21)
Geng 2025 et al.,	NSCLC	-	-	Overexpression	SK-MES-1, H520, H2228, A549	BEAS-2B	(22)
Ma 2023 et al.,	NSCLC	Overexpression	14	Overexpression	A549, H1299, PC9, PC9GR	HBE	(23)
Wu 2023 et al.,	LUAD	-	-	Overexpression	A549, H1299,	BEAS-2B	(24)
Guo 2022 et al.,	LUAD	Overexpression	415	-	-	-	(25)
Tang 2021 et al.,	NSCLC	Overexpression	30	Overexpression	A549, H1299, PC9, PC9GR	HBE	(26)
Zhang 2024 et al.,	NSCLC	Overexpression	40	Overexpression	HCC827, PC-9	BEAS-2B	(27)
Zhang 2022 et al.,	LUAD	Overexpression	55	-	-	-	(28)
Li 2020 et al.,	NSCLC	-	-	Overexpression	LTEP-a-2, H520	HBE	(29)
Xu 2021 et al.,	NSCLC	Overexpression	19	-	-	-	(30)

NSCLC, non-small cell lung cancer; LUAD, lung adenocarcinoma.

**Table 2:** The relationship between the clinicopathological characteristics of NSCLC and KIAA1429 overexpression.

Authors	Type	Prognosis	Clinical indicators	Ref
Guo 2024 et al.,	LUAD	OS	-	(19)
Lin 2023 et al.,	LUAD	-	Pathological stage	(20)
Zhao 2020 et al.,	LUAD	-	Pathological stage, Smoking history, tumor size, lymph node metastasis, distant metastasis	(21)
Ma 2023 et al.,	NSCLC	OS	-	(23)
Guo 2022 et al.,	LUAD	OS	Tumor size, T stage	(25)
Tang 2021 et al.,	NSCLC	OS	Pathological stage	(26)
Zhang 2024 et al.,	NSCLC	-	Pathological stage	(27)

NSCLC, non-small cell lung cancer; LUAD, lung adenocarcinoma; OS, overall survival.

## 3 Molecular functions and signaling mechanisms of KIAA1429

### 3.1 KIAA1429 as an oncogenic driver in NSCLC progression

Accumulating evidence demonstrates that KIAA1429 functions as a critical oncogenic factor in NSCLC pathogenesis. Xu et al., demonstrated that KIAA1429 overexpression significantly enhances the proliferative capacity of multiple NSCLC cell lines, including the A549, PC9, H1299, H1573, H520, PC9GR, HCC827GR, HCC827, and SPCA1 (Table 3). KIAA1429 overexpression was found to accelerate cell cycle transition in LUAD A549 and H1299 cells (Table 3). Ma et al., reported that elevated KIAA1429 expression exerts anti-apoptotic effects in NSCLC PC9GR, HCC827GR, A549, and SPCA1 cells (Table 3). Additionally, Guo et al., revealed that KIAA1429 overexpression promotes the cell migration in NSCLC A549, PC9, H1299, H460, H1573, HCC827GR, PC9GR, HCC827, SPCA1, and H520 cells, and cell invasion in NSCLC A549, PC9, H1573, HCC827GR, H1299, HCC827, SPCA1, and H520 cells (Table 4).

**Table 3:** *In vitro* functional characterization of KIAA1429 in cancer cell growth.

Authors	Type	Cells	Proliferation	Cell cycle	Apoptosis	Ref
Xu 2024 et al.,	LUAD	A549, PC9	Promotion	-	-	(18)
Guo 2024 et al.,	LUAD	A549, H1299	Promotion	-	-	(19)
Lin 2023 et al.,	LUAD	A549, H1573	Promotion	-	-	(20)
Zhao 2020 et al.,	LUAD	A549, H1299	Promotion	Promotion	-	(21)
Geng 2025 et al.,	NSCLC	A549, H520	Promotion	-	-	(22)
Ma 2023 et al.,	NSCLC	PC9GR, HCC827GR	Promotion		Inhibition	(23)
Wu 2023 et al.,	LUAD	A549, H1299	Promotion	-	-	(24)
Tang 2021 et al.,	NSCLC	PC9GR	Promotion	-	-	(26)
Zhang 2024 et al.,	NSCLC	HCC827, PC-9	Promotion	-	-	(27)
Zhang 2022 et al.,	LUAD	A549, SPCA1	Promotion	-	Inhibition	(28)
Xu 2021 et al.,	NSCLC	H520, PC9, A549	Promotion	-	-	(30)

NSCLC, non-small cell lung cancer; LUAD, lung adenocarcinoma.

**Table 4:** *In vitro* functional characterization of KIAA1429 in cancer cell metastasis and drug sensitivity.

Authors	Type	Cells	Migration	Invasion	Gefitinib drug sensitivity	Ref
Xu 2024 et al.,	LUAD	A549, PC9	Promotion	Promotion	-	(18)
Guo 2024 et al.,	LUAD	A549, H1299, H460	Promotion	-	-	(19)
Lin 2023 et al.,	LUAD	A549, H1573, HCC827GR	Promotion	Promotion	Inhibition	(20)
Zhao 2020 et al.,	LUAD	A549, H1299	Promotion	Promotion	-	(21)
Ma 2023 et al.,	NSCLC	PC9GR	Promotion	-	Inhibition	(23)
Wu 2023 et al.,	LUAD	A549, H1299	Promotion	Promotion	-	(24)
Tang 2021 et al.,	NSCLC	PC9GR	Promotion	-	Inhibition	(26)
Zhang 2024 et al.,	NSCLC	HCC827, PC-9	Promotion	Promotion	-	(27)
Zhang 2022 et al.,	LUAD	A549, SPCA1	Promotion	Promotion	-	(28)
Xu 2021 et al.,	NSCLC	H520, PC9, A549	Promotion	Promotion	-	(30)

NSCLC, non-small cell lung cancer; LUAD, lung adenocarcinoma.

Xu et al., further validated these findings using xenograft mouse models, showing that KIAA1429 overexpression enhances tumor growth in NSCLC A549, PC9GR, H1299, and H520 cell-derived tumors (Table 5). These collective findings strongly support the oncogenic role of KIAA1429 in NSCLC, where it drives tumor growth, survival, and metastasis.

**Table 5:** *In vivo* functional characterization of KIAA1429 in NSCLC.

Authors	Type	Cells	Animal model	Role in tumor growth	Ref
Xu 2024 et al.,	LUAD	A549	Male nude mice	Promotion	(18)
Lin 2023 et al.,	LUAD	A549	BALB/c nude mice	Promotion	(20)
Ma 2023 et al.,	NSCLC	PC9GR	Male BALB/c nude mice	Promotion	(23)
Wu 2023 et al.,	LUAD	H1299	Female BALB/c nude mice	Promotion	(24)
Tang 2021 et al.,	NSCLC	PC9GR	Male BALB/c nude mice	Promotion	(26)
Xu 2021 et al.,	NSCLC	H520, A549	BALB/c nude mice	Promotion	(30)

NSCLC, non-small cell lung cancer; LUAD, lung adenocarcinoma.

### 3.2 KIAA1429 as an oncogenic driver in gefitinib resistance of NSCLC

Gefitinib, a first-generation EGFR tyrosine kinase inhibitor (TKI), is a standard targeted therapy for NSCLC harboring EGFR mutations. However, acquired resistance to gefitinib remains a major clinical challenge. Emerging evidence suggests that aberrant expression of KIAA1429 contributes to gefitinib resistance in NSCLC (20, 23, 26). For example, Lin et al., demonstrated that KIAA1429 overexpression promotes gefitinib resistance in HCC827GR and PC9GR cell lines, two established models of acquired TKI resistance (Table 4). These findings highlight the potential therapeutic value of targeting KIAA1429 to restore gefitinib sensitivity in resistant tumors, improve treatment outcomes for NSCLC patients and delay disease progression.

### 3.3 Signaling mechanisms involving KIAA1429 in NSCLC

#### 3.3.1 N6-methyladenosine (m6A)-dependent regulation

As a core component of the m6A methyltransferase complex, KIAA1429 plays a crucial role in maintaining complex stability and directing site-specific m6A modifications through recognition of specific RNA sequences or structural motifs. m6A modification dynamically regulates multiple aspects of RNA metabolism, including splicing, stability, translation, and degradation (36, 37). Current studies have identified that KIAA1429 mediates m6A-dependent regulation of multiple oncogenic targets. Emerging evidence has demonstrated that KIAA1429-mediated m6A modification modulates the expression of multiple downstream targets including LINC01106, ARHGAP30, MAP3K2, MUC3A, KLF1, WTAP, HOXA1, RXFP1, BTG2, and DAPK3, which collectively drive NSCLC progression, metastasis, and the development of gefitinib resistance (Table 6). For example, Zhao et al., demonstrated that KIAA1429 knockdown suppresses MUC3A expression through m6A modification, subsequently inhibiting LUAD cell Proliferation, Cell cycle progression, Migratory capacity and Invasive potential (21). These findings establish KIAA1429 as a master regulator of oncogenic m6A modifications in NSCLC pathogenesis.

**Table 6:** KIAA1429 involves m6A-mediated genes and lncRNA and signaling pathways in NSCLC.

m6A-mediated genes and lncRNA	Signaling pathway	Validated methods	Cancer type	Ref
LINC01106	JAK/STAT3	Western Blotting, RT-PCR, MeRIP-seq	LUAD	(17)
ARHGAP30	-	Western Blotting, MeRIP-seq	LUAD	(18)
MAP3K2	JNK/MAPK	Western Blotting, RT-PCR, MeRIP-seq	LUAD	(19)
MUC3A	-	Western Blotting, RT-PCR, MeRIP-seq	LUAD	(20)
KLF1	PD-L1	Western Blotting, RT-PCR, MeRIP-seq	NSCLC	(21)
WTAP	Autophagy	Western Blotting, RT-PCR, MeRIP-seq	NSCLC	(22)
-	P53, ferroptosis	Western Blotting, ELISA	LUAD	(23)
HOXA1	-	Western Blotting, RT-PCR, MeRIP-seq	NSCLC	(25)
RXFP1	-	Western Blotting, RT-PCR, MeRIP-seq	NSCLC	(26)
BTG2	-	Western Blotting, RT-PCR, MeRIP-seq	LUAD	(27)
DAPK3	-	Western Blotting, RT-PCR, MeRIP-seq	NSCLC	(29)

NSCLC, non-small cell lung cancer; LUAD, lung adenocarcinoma.

#### 3.3.2 Non-m6A dependent signaling pathways in NSCLC

Beyond m6A modification, KIAA1429 promotes tumor growth, metastasis, and gefitinib resistance in NSCLC through multiple signaling pathways including JAK2/STAT3, EMT, PI3K/AKT, PD-L1, autophagy, ferroptosis, p53 signaling pathways (Table 6). Xu et al., revealed that KIAA1429-mediated LINC01106 stabilization activates JAK2/STAT3 signaling by Increasing p-JAK2 and p-STAT3 levels promoting *in vitro* and *in vivo* tumor growth and metastasis (18). Guo et al., reported that KIAA1429 silencing suppresses EMT markers MMP2, ZEB1, β-catenin, and N-cadherin, and Restores E-cadherin expression to inhibit PI3K/AKT activation (19, 21). The multifaceted involvement of KIAA1429 in these pathways highlights its potential as a prognostic biomarker for treatment response.

## 4 Summary and future perspectives

KIAA1429 has emerged as a critical oncogenic regulator in NSCLC, exerting multifaceted roles in tumor progression, metastasis, and drug resistance. KIAA1429 is significantly upregulated in NSCLC tissues and cell lines, correlating with poor prognosis, advanced tumor stage, and metastasis. KIAA1429 promotes cell proliferation, migration, and invasion, and suppresses apoptosis and confers gefitinib resistance using m6A-dependent RNA methylation, influencing key oncogenes/tumor suppressors (such as the LINC01106, MUC3A, BTG2) and non-m6A pathways: Activates JAK2/STAT3, PI3K/AKT, PD-L1, and disrupts p53, autophagy, and ferroptosis. Despite these advances, several key questions remain. How does KIAA1429 selectively target specific RNAs for m6A modification? Are there tissue-specific or mutation-dependent regulatory networks? Can KIAA1429 inhibition synergize with existing therapies (such as the EGFR-TKIs)? KIAA1429 represents a promising therapeutic target and prognostic marker in NSCLC. Future studies should focus on elucidating its precise mechanisms, developing targeted inhibitors, and validating its clinical utility. Addressing these challenges may open new avenues for overcoming drug resistance and improving NSCLC treatment outcomes.
